# Supplementary figures and images for: The Aspergillus nidulans velvet domain containing transcription factor VeA is shuttled from cytoplasm into nucleus during vegetative growth and stays there for sexual development, but has to return into cytoplasm for asexual development
Source: PLoS Genet. 2025 Jun 16;21(6):e1011687. doi: 10.1371/journal.pgen.1011687 (PMC12169562; doi:10.1371/journal.pgen.1011687)

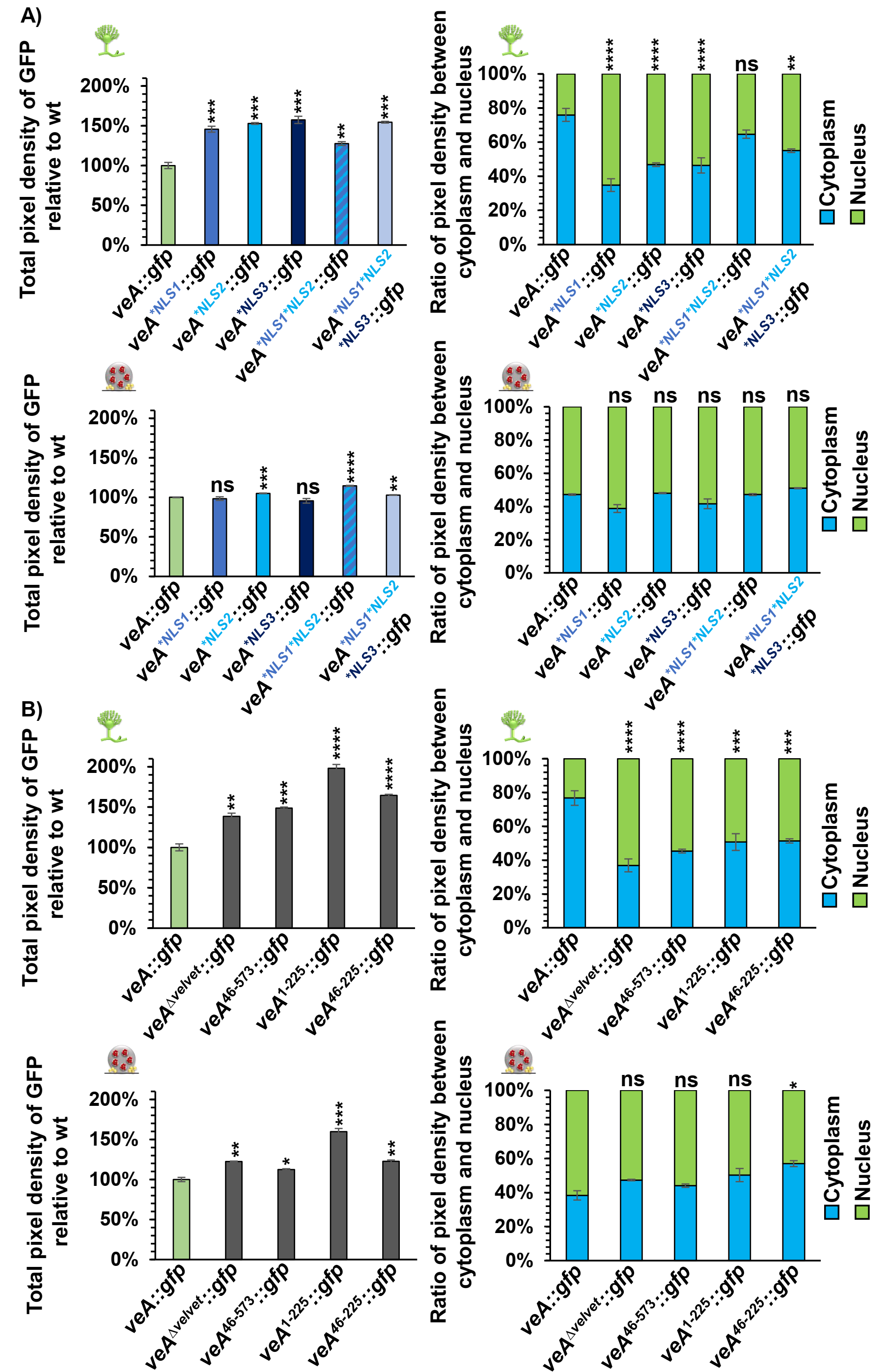

Supplement: S1 Fig — (A) and (B) shows quantification of total GFP-signals as well as the ratio of GFP-signals in cytoplasm (turquoise) and nuclei (green) produced under illuminating conditions inducing asexual and in the dark inducing sexual development (71–108 nuclei for each of three biological replicates). Error bars for quantification represent standard error of the mean (p > 0.05: ns, p ≤ 0.05: *, p ≤ 0.01: **, p ≤ 0.001: ***, p ≤ 0.0001: ****). (TIF) [file pgen.1011687.s001.tif]

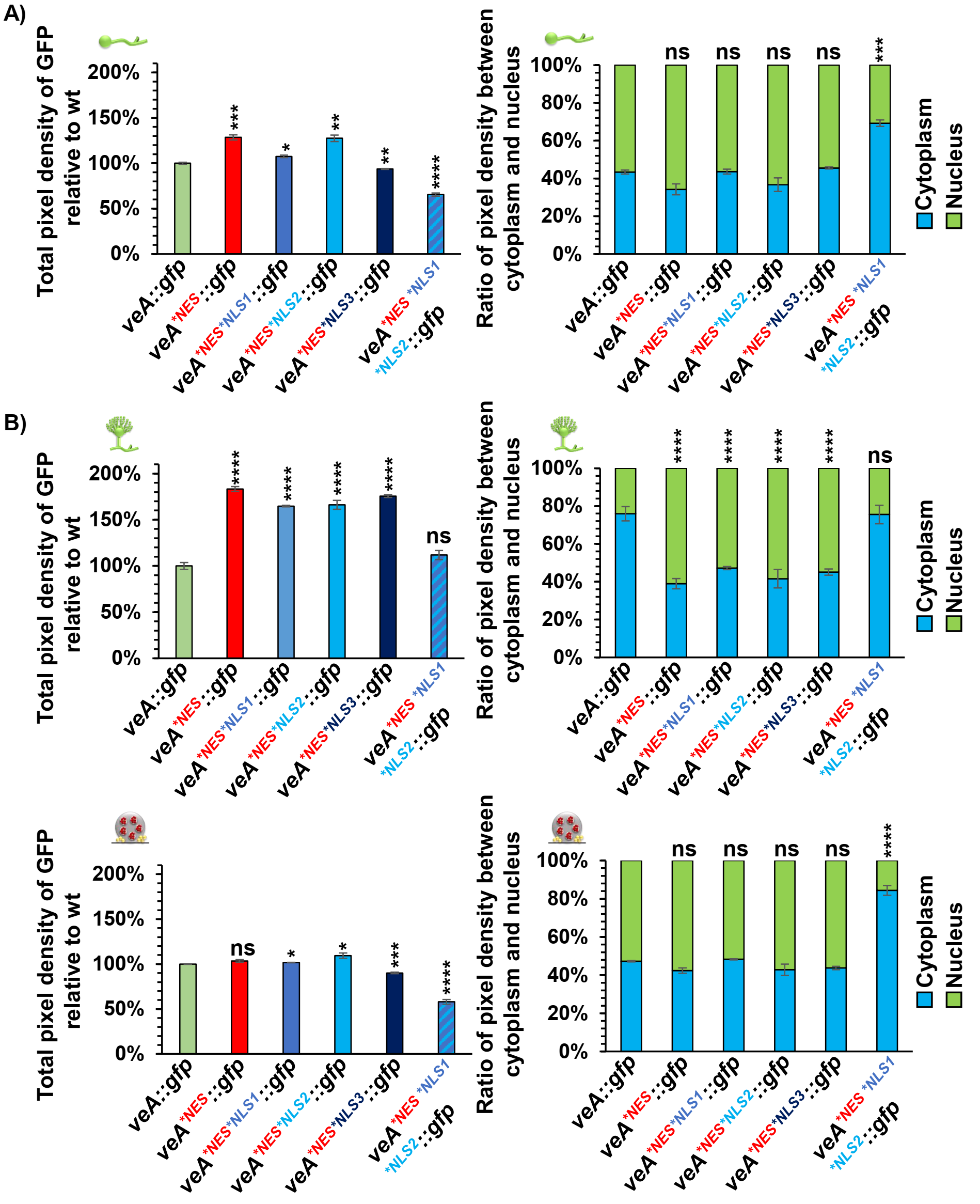

Supplement: S2 Fig — Total GFP-signals as well as the ratio of GFP-signals in in cytoplasm (turquoise) and nuclei (green) produced under vegetative conditions (A) as well as illuminating conditions inducing asexual and in the dark inducing sexual development (B) were quantified (45–100 nuclei for each of three biological replicates). Error bars for quantification represent standard error of the mean (p > 0.05: ns, p ≤ 0.05: *, p ≤ 0.01: **, p ≤ 0.001: ***, p ≤ 0.0001: ****). (TIF) [file pgen.1011687.s002.tif]

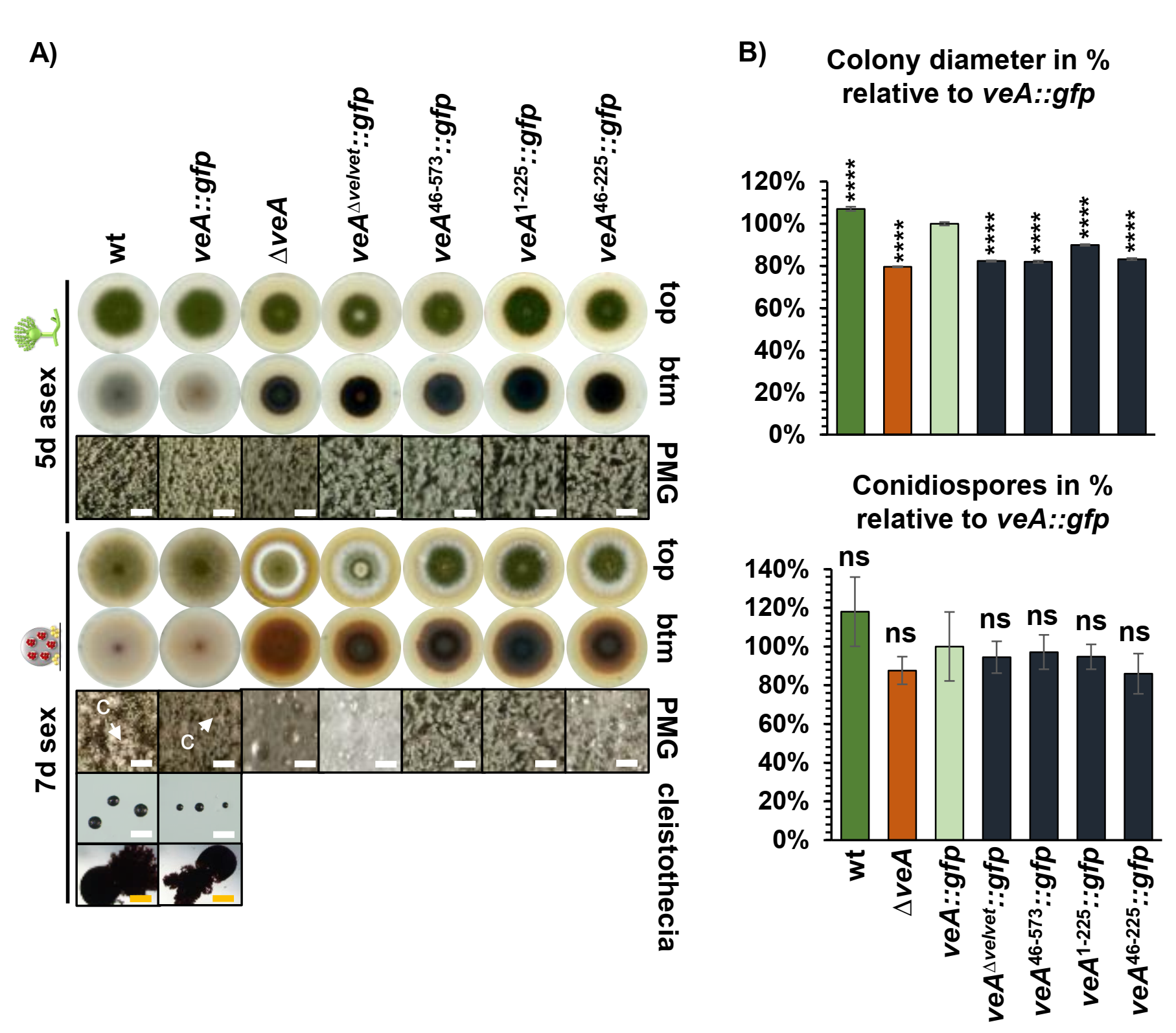

Supplement: S3 Fig — (A) Asexual development of veA deletion (ΔveA) and complementation (veA::gfp) strains as well as truncated versions of VeA (veAΔvelvet::gfp, veA46-573::gfp, veA1-225::gfp, veA46-225::gfp) were analyzed after five days incubation at 37°C in light. Plates were incubated in the dark and with limited oxygen for seven days at 37°C for sexual development. The dark brownish color of the veA deletion strain at the bottom of the plate indicates alterations in secondary metabolism combined with failure to form cleistothecia as defects in sexual development. Strains with truncated versions of VeA resembled the veA deletion phenotype to a certain degree. The truncated version having a full or a truncated velvet domain produce more spores and less aerial hyphae compared to the veA deletion phenotype (PMG: photomicrographs, c: cleistothecia, btm: bottom, size bar: 200 µm, cleistothecia 50 µm). (B) Quantification of colony diameters and spore amounts from wildtype, veA deletion and veA truncations. Colony diameters of strains with truncated VeA have a significantly similar reduction compared to veA::gfp. This is also the case for the spore production of mutant strains compared to veA::gfp, whereas the tendency shows no significance. In total, this supports that the full VeA protein is required for spore production during asexual development. Quantification results from three biological replicates (3 technical replicates each). Error bars represent standard error of the mean, and the significances were compared to veA::gfp complementation strain (p > 0.05: ns, p ≤ 0.0001: ****). (TIF) [file pgen.1011687.s003.tif]

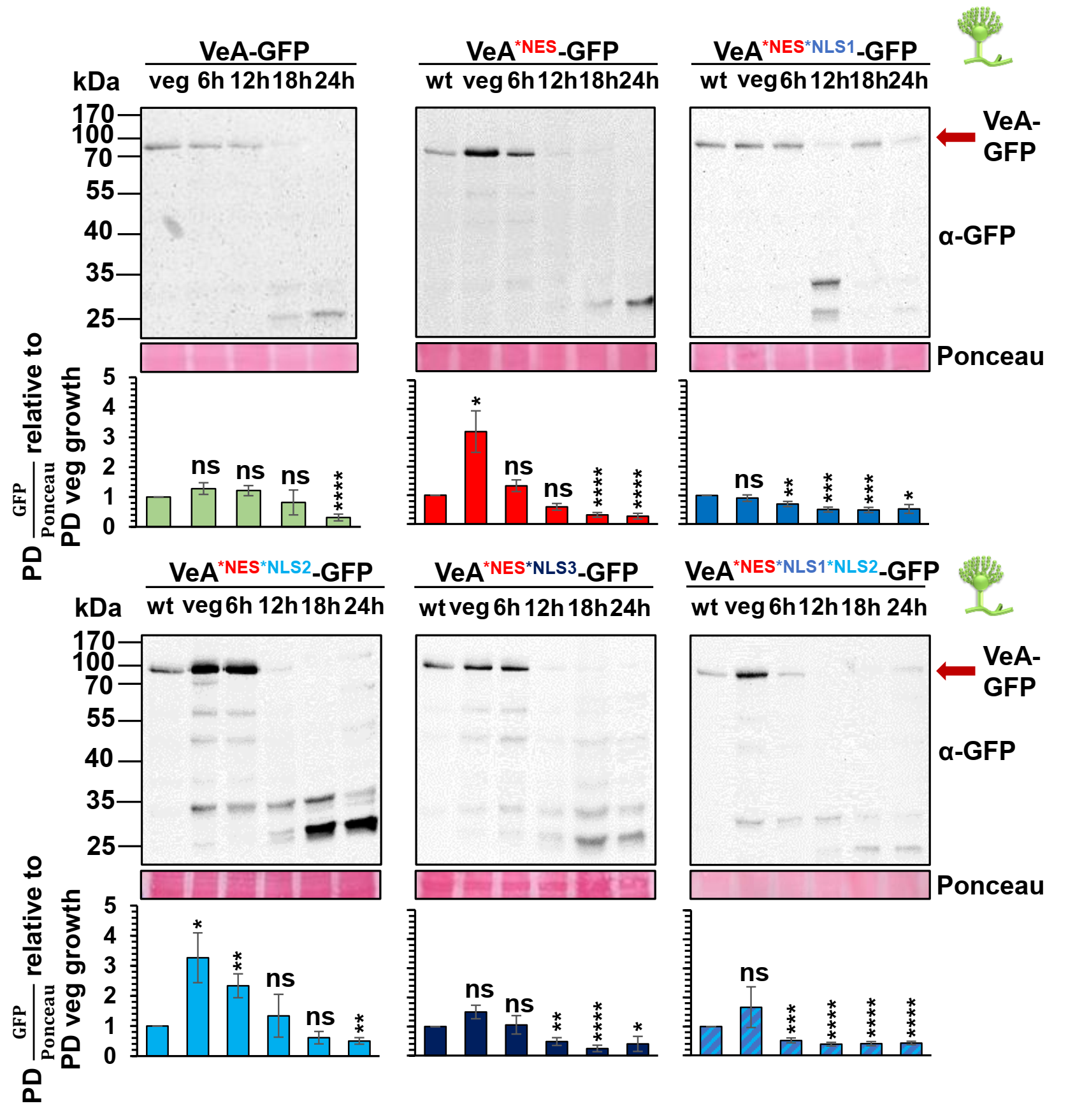

Supplement: S4 Fig — Western hybridization of fungal protein extracts from VeA and VeAs complementation strains with amino acid substitutions in NES and NLS motifs were performed. All strains were grown for 20 h at 37°C in liquid minimal medium to enable vegetative growth. The mycelia were then shifted onto 30 ml minimal medium plates for 6–24 h in light at 37°C for asexual development. Samples were collected at identical time points and protein crude extracts were prepared for western hybridization with α-GFP antibody. Signal quantification was performed using BioID software, signals of VeA-GFP (red arrow) were normalized to Ponceau staining. The wild type (wt) VeA protein amount at the 20 h vegetative time point was set to 1 and used to compare the protein amount of following time points of wild type VeA as well as VeA with amino acid substitutions in the NES and NLS motifs. Quantification results from three biological replicates (2 technical replicates each). Error bars for western hybridization represents standard error of the mean the significances were compared to vegetative growth of each strains respectively (p > 0.05: ns, p ≤ 0.05: *, p ≤ 0.01: **, p ≤ 0.001: ***, p ≤ 0.0001: ****). (TIF) [file pgen.1011687.s004.tif]

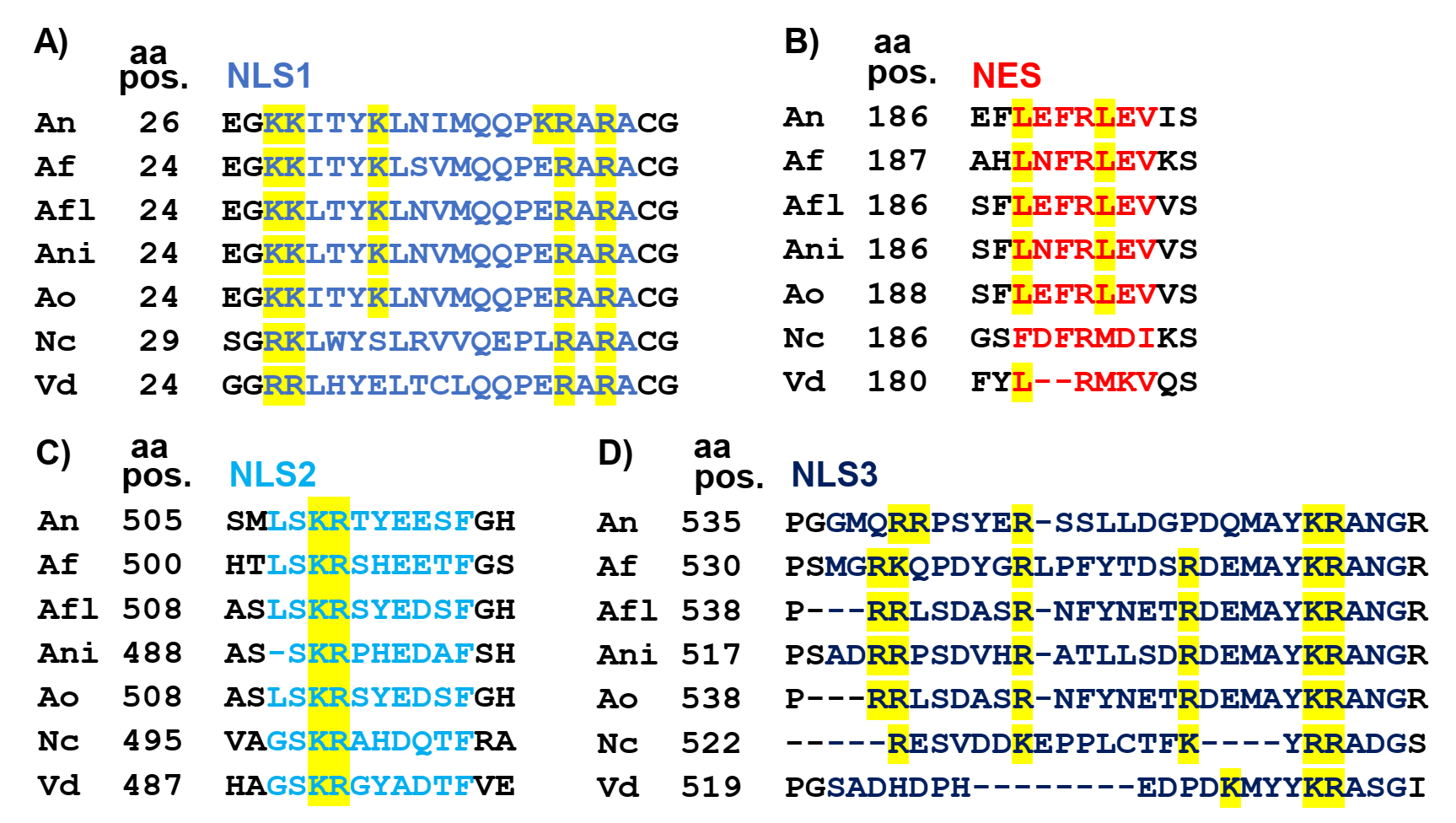

Supplement: S5 Fig — Clustal Omega protein sequence alignments of the three NLS and the NES were performed between fungal VeA orthologs. The NLS1 (blue), NLS2 (turquoise) and NLS3 (dark blue) motifs are conserved in A. nidulans (An), A. fumigatus (Af), A. flavus (Afl), A. niger (Ani), A. oryzae (Ao) and N. crassa (Nc). The NLS3 motif is not conserved in V. dahliae (Vd). The NES motif is only conserved in Aspergillus species but not in N. crassa or V. dahliae. Conserved lysine (K), arginine (R), and leucine (L) residues within the consensus sequences are marked in yellow. (TIF) [file pgen.1011687.s005.tif]
