# Supplementary material for: The Aspergillus nidulans velvet domain containing transcription factor VeA is shuttled from cytoplasm into nucleus during vegetative growth and stays there for sexual development, but has to return into cytoplasm for asexual development
Source: PLoS Genet. 2025 Jun 16;21(6):e1011687. doi: 10.1371/journal.pgen.1011687 (PMC12169562; doi:10.1371/journal.pgen.1011687)
Supplement: S1 Table — (DOCX) [file pgen.1011687.s006.docx]

S1_Table

| **No.** | **Chemical name** | **Molecular Formula** | **Calc. exact mass [M]** | **Measured exact mass [M+H]^+^** | **Rt (min)** | **Confirmed by** | **Reference** | **Gene cluster** |
| --- | --- | --- | --- | --- | --- | --- | --- | --- |
| I | Cichorine | C_10_H_11_NO_3_ | 193.073894 | 194.0814 | 7.6 | A, C | (1) | cic, CicF (nrPKS, AN6448) |
| II | F-9775A/B | C_21_H_16_O_8_ | 396.08452 | 397.0917 | 9.5,  10.5 | A, C | (2) | ors, OrsA (nrPKS, AN7909) |
| III | Austinol | C_25_H_40_O_8_ | 458.19407 | 459.2012 | 12.7 | A, B | (3) | aus, AusA (nrPKS, AN8383) |
| IV | Dehydroaustinol | C_25_H_28_O_8_ | 456.17842 | 457.1853 | 13.1 | A, B | (3) | aus, AusA (nrPKS, AN8383) |
| V | Sterigmatocystin | C_18_H_12_O_6_ | 324.06339 | 325.0703 | 15.3 | A,B | (4) | stc, StcA (nrPKS, AN7825) |
| VI | Emericellin | C_25_H_28_O_5_ | 408.193675 | 409.2005 391.1898  [M-H_2_O+H]^+^ | 24.6 | A, B | (5) | mdp, mdpG (nrPKS, AN0150) |
| VII | Shamixanthone | C_25_H_26_O_5_ | 406.178025 | 389.1743  [M-H_2_O+H]^+^ | 24.8 | A, B | (5) | mdp, MdpG (nrPKS, AN0150) |
| VIII | Epishamixanthone | C_25_H_26_O_5_ | 406.178025 | 389.1741  [M-H_2_O+H]^+^ | 25.8 | A, B | (5) | mdp, MdpG (nrPKS, AN0150) |

Peak numbers from CAD and EIC (Fig 4, Fig 5) correspond to secondary metabolites. A: Exact mass measurement, B: retention time (6,7)/ comparison with commercial standards for sterigmatocystin, C: UV/VIS spectrum (1,2).

**References**

1. Sanchez JF, Entwistle R, Corcoran D, Oakley BR, Wang CCC. Identification and molecular genetic analysis of the cichorine gene cluster in *Aspergillus nidulans*. Med Chem Commun. 2012;3:997–1002.

2. Bok JW, Chiang YM, Szewczyk E, Reyes-Domingez Y, Davidson AD, Sanchez JF, et al. Chromatin-level regulation of biosynthetic gene clusters. Nat Chem Biol. 2009;5(7):462–4.

3. Lo H-C, Entwistle R, Guo C-J, Ahuja M, Szewczyk E, Hung J-H, et al. Two separate gene clusters encode the biosynthetic pathway for the meroterpenoids, austinol and dehydroaustinol in *Aspergillus nidulans*. J Am Chem Soc. 2012;134(10):4709–20.

4. Yu JH, Leonard TJ. Sterigmatocystin biosynthesis in *Aspergillus nidulans* requires a novel type I polyketide synthase. J Bacteriol. 1995;177(16):4792–800.

5. Sanchez JF, Entwistle R, Hung J-H, Yaegashi J, Jain S, Chiang Y-M, et al. Genome-Based Deletion Analysis Reveals the Prenyl Xanthone Biosynthesis Pathway in *Aspergillus nidulans*. J Am Chem Soc [Internet]. 2011;133(11):4010–7.

6. Liu L, Sasse C, Dirnberger B, Valerius O, Fekete-Szücs E, Harting R, et al. Secondary metabolites of hülle cells mediate protection of fungal reproductive and overwintering structures against fungivorous animals. Elife. 2021;10:1–28.

7. Thieme KG, Gerke J, Sasse C, Valerius O, Thieme S, Karimi R, et al. Velvet domain protein VosA represses the zinc cluster transcription factor SclB regulatory network for *Aspergillus nidulans* asexual development , oxidative stress response and secondary metabolism. PLoS Genet. 2018;14(7):1–45.
