# Supplementary material for: The Aspergillus nidulans velvet domain containing transcription factor VeA is shuttled from cytoplasm into nucleus during vegetative growth and stays there for sexual development, but has to return into cytoplasm for asexual development
Source: PLoS Genet. 2025 Jun 16;21(6):e1011687. doi: 10.1371/journal.pgen.1011687 (PMC12169562; doi:10.1371/journal.pgen.1011687)
Supplement: S5 Table — (DOCX) [file pgen.1011687.s010.docx]

S5_Table

| Primer | Sequence | Reference |
| --- | --- | --- |
| JG814 | CAACGAGCATCAGCACAAACAT | Dr. J. Gerke, p.c |
| JG815 | ATCTTCTACGCCGATTCCTGCT | Dr. J. Gerke, p.c |
| JG880 | ACGACGAGGGTCTTATTGTCA | Dr. J. Gerke, p.c |
| JG881 | CCTTCTCCTGGGTAGTGAA | Dr. J. Gerke, p.c |
| MG277 | CGTCTTCTTCGCAAGGGAAACT | Dr. M. Gulko, p.c |
| MG278 | CGGGTTTTCTTGTTGTCACGAG | Dr. M. Gulko, p.c |
