# Supplementary material for: The Aspergillus nidulans velvet domain containing transcription factor VeA is shuttled from cytoplasm into nucleus during vegetative growth and stays there for sexual development, but has to return into cytoplasm for asexual development
Source: PLoS Genet. 2025 Jun 16;21(6):e1011687. doi: 10.1371/journal.pgen.1011687 (PMC12169562; doi:10.1371/journal.pgen.1011687)
Supplement: S6 Table — (DOCX) [file pgen.1011687.s011.docx]

S6_Table

|  | **Velvet domain of VeA** |
| --- | --- |
| **PDB id** | 9I2S |
| **Wavelength [Å]** | 0.8 |
| **Resolution range [Å]** | 47.21    - 2.4 (2.486    - 2.4) |
| **Space group** | P 2(1) 2(1) 2 |
| **Unit cell [Å, °]** | 106.04 156.08 52.73 90 90 90 |
| **Total reflections** | 144071 (15106) |
| **Unique reflections** | 34475 (3372) |
| **Multiplicity** | 4.2 (4.4) |
| **Completeness (%)** | 98.00 (98.2) |
| **Mean I/sigma(I)** | 22.93 (1.05) |
| **Wilson B-factor [Å^2^]** | 71.49 |
| **R-merge** | 0.041 (1.821) |
| **R-meas** | 0.0470 (2.078) |
| **R-pim** | 0.02504 (1.008) |
| **CC1/2** | 1 (0.504) |
| **CC*** | 1 (0.819) |
| **Reflections used in refinement** | 34383 (3370) |
| **Reflections used for R-free** | 1719 (169) |
| **R-work** | 0.2193 (0.4096) |
| **R-free** | 0.2531 (0.4813) |
| **CC(work)** | 0.957 (0.499) |
| **CC(free)** | 0.963 (0.316) |
| **Number of non-hydrogen atoms** | 5530 |
| **macromolecules** | 5486 |
| **ligands** | 0 |
| **solvent** | 44 |
| **Protein residues** | 682 |
| **RMS (bonds) [Å]** | 0.004 |
| **RMS (angles) [°]** | 0.70 |
| **Ramachandran favored (%)** | 97.87 |
| **Ramachandran allowed (%)** | 2.13 |
| **Ramachandran outliers (%)** | 0.00 |
| **Rotamer outliers (%)** | 1.80 |
| **Clashscore** | 5.78 |
| **Average B-factor [Å^2^]** | 102.05 |
| **macromolecules** | 102.28 |
| **solvent** | 74.53 |
| **Number of TLS groups** | 26 |

Statistics for the highest-resolution shell are shown in parentheses.
