# Supplementary material for: The Aspergillus nidulans velvet domain containing transcription factor VeA is shuttled from cytoplasm into nucleus during vegetative growth and stays there for sexual development, but has to return into cytoplasm for asexual development
Source: PLoS Genet. 2025 Jun 16;21(6):e1011687. doi: 10.1371/journal.pgen.1011687 (PMC12169562; doi:10.1371/journal.pgen.1011687)
Supplement: Attachment — Submitted filename: Response to Reviewers.docx [file pgen.1011687.s013.docx]

Dear Editors,

We have addressed all comments and suggestions as follows:

1) Please include a completed 'Competing Interests' statement in the text box when submitting your production task, including any COIs declared by your co-authors, written in full sentences.
If you have no competing interests to declare, please state "The authors have declared that no competing interests exist". You may also provide an updated statement via email.

“The authors have declared that no competing interests exist” was added to the statement box.

2) Please ensure that the affiliations of the authors listed on the manuscript title page do exactly match with the affiliations provided in the online submission form
NOTE: Affiliations should include a department (if applicable), an institution, a CITY, and a country
The affiliations were corrected in the online form and on the manuscript the city and country was added.

3) We do not publish any copyright or trademark symbols that usually accompany proprietary names, eg ©,  ®, or TM  (e.g. next to drug or reagent names). Therefore please remove all instances of trademark/copyright symbols throughout the text, including:  qPCR™ on page no 30, Manager™ on page no 31, and QuikChange(TM) on page no 36.
The mentioned trademarks were corrected in the manuscript.

4)  Please ensure that the funders and grant numbers match between the Financial Disclosure field and the Funding Information tab in your submission form. Note that the funders must be provided in the same order in both places as well.
Currently, " IRTG PRoTECT" is missing from the Funding Information

"IRTG PRoTECT" was added to the funding information.

We ask that a manuscript source file is provided at Revision. Please upload your manuscript file as a .doc, .docx, .rtf or .tex. If you are providing a .tex file, please upload it under the item type u2018LaTeX Source Fileu2019 and leave your .pdf version as the item type u2018Manuscriptu2019.

- The manuscript has been uploaded as word document.

We do not publish any copyright or trademark symbols that usually accompany proprietary names, eg ©,  ®, or TM  (e.g. next to drug or reagent names). Therefore please remove all instances of trademark/copyright symbols throughout the text, including:
- ® on pages: 23, 28, and 29
- TM on pages: 29, and 30.

- All symbols have been removed as suggested.

Please upload all main figures as separate Figure files in .tif or .eps format. For more information about how to convert and format your figure files please see our guidelines: 
<https://journals.plos.org/plosgenetics/s/figures>

- All figures have been uploaded separately.

Some material included in your submission may be copyrighted. According to PLOSu2019s copyright policy, authors who use figures or other material (e.g., graphics, clipart, maps) from another author or copyright holder must demonstrate or obtain permission to publish this material under the Creative Commons Attribution 4.0 International (CC BY 4.0) License used by PLOS journals. Please closely review the details of PLOSu2019s copyright requirements here: PLOS Licenses and Copyright. If you need to request permissions from a copyright holder, you may use PLOS's Copyright Content Permission form. Please respond directly to this email and provide any known details concerning your material's license terms and permissions required for reuse, even if you have not yet obtained copyright permissions or are unsure of your material's copyright compatibility. Once you have responded and addressed all other outstanding technical requirements, you may resubmit your manuscript within Editorial Manager. 
Potential Copyright Issues:
i) Figures 2(B, C), 3, 4(A, C), 5(A, C)6, 7, S1, S2, S3A, and S4. Please confirm whether you drew the images / clip-art within the figure panels by hand. If you did not draw the images, please provide (a) a link to the source of the images or icons and their license / terms of use; or (b) written permission from the copyright holder to publish the images or icons under our CC BY 4.0 license. Alternatively, you may replace the images with open source alternatives. See these open source resources you may use to replace images / clip-art:
- https://commons.wikimedia.org
- <https://openclipart.org/>.

- All presented cliparts were created by the first author Anja Strohdiek by PowerPoint.

Thank you for stating "The data for the crystal structure is available in the RCSB Protein Data Bank accessable with the code 9I2". Please note that, though access restrictions are acceptable now, your entire minimal dataset will need to be made freely accessible if your manuscript is accepted for publication. This policy applies to all data except where public deposition would breach compliance with the protocol approved by your research ethics board. If you are unable to adhere to our open data policy, please kindly revise your statement to explain your reasoning and we will seek the editor's input on an exemption.

-The crystal structure is after acceptance of the manuscript freely available on the RCSB Protein Data Bank. The code 9I2S is required to find the structure on the data bank. The validation report of PDB is also uploaded.

Please ensure that the funders and grant numbers match between the Financial Disclosure field and the Funding Information tab in your submission form. Note that the funders must be provided in the same order in both places as well. "Open Access Publication Funds of the University of Göttingen" is missing from the Funding Information tab.

"Open Access Publication Funds of the University of Göttingen" was changed to the acknowledgment, since this is not a grand of the research group.

**Reviewer #1**

Velvet proteins are key regulators of fungal sexual and asexual development and secondary metabolism. The most well-studied velvet protein is VeA (Velvet A), which plays a crucial role in coordinating fungal growth, reproduction, and the production of secondary metabolites, including toxins and antibiotics. veA protein from Aspergillus nidulans is the most intensively studied one, and the manuscript provides significant answers to the important question about the regulatory role of veA in fungal development. The authors show how a complex shuttle control mechanism for VeA localization either in the nucleus or in cytoplasm is controlling different fungal developmental processes.
The precise nuclear import and export control of velvet proteins is a prerequisite for fungal development and secondary metabolism.
After prediction of the crystal structure of the N-terminal VeA protein, including the velvet domain, the authors provide an extensive functional analysis using different truncated, as well as mutated versions of the veA gene. They use mutants, expressing genes for different NLS, NES variants. For example, NLS motifs are required for asexual development, or for conidiospore or cleistothecia maturation. Similarly, NES allows the coordinated development and secondary metabolism. These analyses are an important part of this manuscript.

My only criticism concerns the designation of the mutant constructs in different analysis. Probably, I had some difficult to follow these investigations, because the NLS and NES mutants are not precisely/unambiguously described. For example, VeA*NES*NLS1*NLS2*NLS3 and the corresponding plasmids are only mentioned at the end of the manuscript (line 687ff). It would be further very helpful to see the different combinations with the amino acid substitutions of the NLS elements (line 88 ff). In Fig. 1A, these substitutions are displayed, however in the text, I miss the corresponding references. An error in Fig. 1A let the reader suggest that two variants are shown for NLS1. Indeed, the “KR” amino acids are not at position 44/45 (Fig.1A), but instead at position 41/42. This mistake implies that two different AA substitutions were constructed. I suggest mentioning the nomenclatures NLS* etc in Fig. 1A. Table S2 contains the correct positions.

The mentioned plasmid was used to as precursor to generate the plasmid, which has then been transformed into *Aspergillus*. This has now been clarified in the methods section (line 700-711). The missing reference for Fig 1A was added in line 143-146. The error in Fig 1A was corrected. The KR Position of 45 was changed to 42.

Suggested changes and minor points:

Line 178 “…….addition to the veA:gfp fusion genes with point mutated codons for the signal sequences (Fig 2A).” (I see the mutated codons only in Fig. 1A.)

Fig 2A refers to the truncated versions. Fig 1A is now added to refer to the altered codons (line 190).

192 ff: changes and additional combinations with the amino acid substitutions of the NLS elements described before….. (indicate that it is only mentioned in the legend of Fig. 1A.)

The reference Fig 1A was added to indicate the changes (line 204).

Line 63: be more specific “…. fungus starts with asco- or conidiospore germination…..”

We changed as suggested (line 64).

Line 426 delete “an”, “Taken together, an all tested…”

Changed as suggested.

Formal comment: Give all species names in the reference titles in italics, e.g. reference 36, 37, 42 (even though the Journal is not consistent in diverse papers).

We changed all species names to italic.

**Reviewer #2:**

The manuscript by Strohdiek et al describes the protein elements that are required for the correct cellular localization of the veA protein, a component of the velvet complex, in the fungus Aspergillus nidulans. The authors identified three nuclear localization signals (NLS) and one nuclear exclusion signal (NES) in VeA, and have created a series of single, and multiple mutations in each of the domains to assay their role in the nuclear localization of the protein during sexual and asexual development, and their role in the accumulation of secondary metabolites. The authors have also characterized the transcription of each mutant gene and the stability of the wild type and mutant proteins during development. The authors have shown that the three NLS domains and the NES act in coordination to regulate the changes in the localization of VeA in the fungal cell during sexual and asexual development. The balance of sexual and asexual development requires a correct cellular localization of VeA and its interaction with other velvet proteins, and the authors have shown that the NLSs and NEs in VeA are required for the correct localization and stability of VeA during the regulation of development and secondary metabolism. The manuscript is well written and the results support the conclusions of the manuscript. I have several comments that I hope will improve the final manuscript

Figure 1 legend. A. "Functional studies of all NLS and the NES sequences were conducted by codon exchanges for the indicated amino acids.". From the figure I think that the authors refer to the amino acids shown below each domain. Please, indicate what are the wt amino acids and the mutant amino acids. Also, explain what are the amino acids shown in the box below the protein scheme. I assume that they are the full amino acid sequences for each domail but I suggest that this is indicated clearly in the legend and in the figure.

In the figure 1 wt and mt was added to indicate which amino acids correspond to the wildtype (wt) versions and which the mutant (mt) versions. In the box below it was added, that the amino acids belongs to the wild type motifs. Accordingly, the legend was modified.

2. Results. Page 6. Lines 146-147. “NLS1-3 cooperate in VeA nuclear import and are required to exclude the protein from the nucleus in light when asexual development is promoted”. I suggest that the authors clearly state the growth conditions, including illumination conditions, for the experiments that they plan to perform right at the start of this section. The information is described in materials and methods and also later in the text but I suggest that they provide a clear description at the start for the sake of clarity. Sometimes the authors refer to growth conditions as under light or dark, in others, they specify the growth conditions are promoting sexual versus asexual development. Also the illumination conditions in vegetative growth should be described as well.

More information was added to the growth conditions in the result section to clarify the different conditions (line 153- 157). More information was also added to the corresponding fig 2 (line 171-173).

Figure 2 legend and figures. A. Please, in the legend replace “Schema” by “Scheme”. Please, indicate in the figure that the numbers above the first scheme correspond to the amino acids of the protein. In the mRNA, please, indicate in the mRNA scheme the presence of an intron. In the figure, the kDa above the VeA delta velvet presumably indicate the molecular weight of each segment of the fused protein. Please, indicate the kDa for the other protein fusions. If the authors consider that this is too much information for the figure, please, consider deleting the MW for this specific fusion. B. Please, indicate when the cultures were kept in light or in dark with L or D in the figure, in addition to indicating the vegetative or developmental program. C. Please, indicate in the legend the conditions for vegetative growth (liquid culture with shaking?, dark or light?).

‘Schema’ was replaced by Scheme as suggested. More details about the intron were added in Fig 2. The term kDa was placed above the full length VeA to indicate that the kDa belongs to the full length VeA. The kDa for the variants were not added to avoid too much information in the figure. For C: The conditions of the vegetative growth was added: illuminated liquid culture. No shaking occurred here, the strains were incubated in inoculated in 400 µl liquid MM in 8‑well microscope chambers (Ibidi GmbH) (see method section). For B: We used the corresponding fruiting bodies, cleistothecia and conidiophore in the description of the legend to indicate the growth condition during light or darkness. Hyphae in the legend represents vegetative growth. We clarified this now more in the legends for Fig 2 and 3.

Results. Page 7. Line 169. “increased nuclear accumulation in light of approximately 30%...” Since the quantifications are shown in fig. S1 I suggest indicating this supplementary figure after this statement.

Fig S1 was added as reference (l 180).

Results. Page 7. Line 178. Gene fusions are usually shown with the symbol “::” between the two fused segments. Please, correct.

Corrected as suggested.

Legend to figure S1. Lines 981-983. Please correct GPF to GFP and delete the double “in”.

Corrected as suggested.

Legend to figure 4. Please, do not include results in the legend (for example, lines 260-266). Idem for legend to figure 5.

Results are removed from the legends as suggested.

Discussion. Mutations in NLS and/or NES result in nuclear localization of VeA during asexual growth (light), however, the amount of conidia in these mutant strains is similar to that obtained from the wild type (figures 4-5). The authors should discuss how these contradictory results could be explained under the hypothesis that VeA should be excluded from the nuclei to allow asexual development.

We assume that the interplay between NLS3 and the NES plays a specific role for asexual development and conidia formation. VeA might co-transport other yet unknown interaction partners by dimer formation in or outside the nucleus. This might play a decisive role in controlling fungal development and should be addressed in future studies. We added this to line 464 ff.

Discussion. Can the results obtained in this manuscript be extended to the activity of other VeA homologs? I think that the text will improve if the authors could include a discussion on the presence of NLS or NES in other VeA homologs, at least in the homologs that have been described in more detail such as in N. crassa or Fusarium. Also, I suggest that the authors could incorporate in the discussion what could be consequence of the export of VeA from the nuclei in the interaction of VeA with the other velvet proteins and the formation of regulatory complexes. Could some of the phenotypes observed be a consequence of diferential interaction with other velvet proteins in the NLS or NES mutants?

We have extended the discussion as suggested by an alignment of the motifs with other related *Aspergillus* species as well as with organisms like *Verticillium dahliae* and *Neurospora crassa*. This alignment of the VeA motifs by Clustal Omega protein sequence alignment shows that NLS1 and NLS2 are conserved between *Aspergillus* species, *Verticillium dahliae* as well as *Neurospora crassa*. The NES motifs also are conserved in different *Aspergillus* species, but is neither present in *N. crassa* or *V. dahliae*. NLS3 is conserved in Aspergilli. We have added supplementary figure Fig S5, showing these alignments, which is described in lines 543 ff. Figure legend is on page 39/40, line 1037-1043. Additionally, we added in the discussion, that the defective motifs could lead to a disturbance of the VeA-VelB heterodimer formation as well as of the velvet complex with LaeA. We discussed (i) whether the inability to form such complexes resulted in the observed phenotypical effect or (ii) the observed phenotype might be the consequence of defective nuclear export of VeA (lines 498 ff).

**Reviewer #3:**

The heterotrimeric velvet-complex VelB/VeA/LaeA is the central regulator of sexual and asexual morphogenesis and secondary metabolism in fungi. In Aspergillus nidulans, the localisation of the transcription factor VeA depends on light. In the dark, VeA is imported to the nucleus to allow sexual development, while light induces cytosolic localisation and the asexual pathway. The authors investigated in their work the role of the three nuclear localisation signals (NLS1-NLS3) and the nuclear export sequence (NES) for the localisation of VeA. This was achieved by the construction of various VeA-GFP fusion proteins with point mutations in NLS1, NLS2, NLS3 and NES. In addition, truncated versions of VeA fused to GFP were constructed. These experiments revealed that VeA is already localised in the nucleus during vegetative growth. Furthermore, the combination of NLS1, NLS2 and NES motifs seems to be required for the nuclear import of VeA. In contrast, NLS3 probably cooperates with NES to induce cytoplasmic localisation and asexual spore production. Cytoplasmic localisation of VeA seems to prevent its degradation and stabilises the protein. The authors also determined the crystal structure of the N-terminal part of VeA, which illustrated the spatial localisation of the three NLS and the NES-motif. In addition, the authors showed that the transition to either sexual or asexual development in dependence of the VeA localisation went along with the production of specific secondary metabolites, which was detected by LC-MS-analysis. In summary, the manuscript demonstrates the complex regulation of the VeA localisation for coordinating fungal development

The authors should emphasise more strongly which of their findings are new and how they can complete the picture of developmental regulation in fungi. For example, the abstract needs improvement to summarise the author’s experimental work and reads more like an abstract for a review article

The abstract was modified as suggested.

VeA orthologues exist in many fungi, for some it was shown that they are either required for infections of plants or humans, e.g. Aspergillus, Fusarium, Cochliobolus and Magnaporthe. Can the author’s findings be discussed in the context of other fungi? How conserved are the NLS and NES motifs in such fungi? The authors should address these aspects in their manuscript.

We added into the discussion an alignment of the motifs with other related *Aspergillus* species as well as with organisms like *Verticillium dahliae* and *Neurospora crassa*. An alignment of the VeA motifs by Clustal Omega protein sequence alignment shows that NLS1 and NLS2 are conserved in *Aspergillus* species, *Verticillium dahliae* and *Neurospora crassa*. The NES motif seems to be conserved in these *Aspergillus* species since they share the amino acid sequence. The NES is not present in either *N. crassa* or *V. dahliae*. The alignment of the NLS3 shows that the sequence might be conserved in Aspergilli. Added to line 543 ff. Additionally, we added another supplementary figure (Fig S5), showing these alignments. Figure legend on page 39/40, line 1037-1043.

The localisation of VeA is also influenced by its binding to other proteins. For example, importin KapA recognises the VeA-VelB dimer for nuclear import or the LeaA-like methyltransferase LlmF has an impact on the localisation of VeA. I recommend the authors shortly discuss how the interaction of VeA with other proteins could influence the accessibility to the NLS or NES motifs.

The changing of one or more motifs could lead to a change in the structure of the protein and thus make it more difficult or easier for VeA to bind to interaction partners. We added this in the discussion section line 515 ff.

Minor points

p. 11, line 290: Shortly mention the amino acid substitutions.

The amino acid substitution was added in line 293.

p. 23, line 582: Windows error message should be deleted.

We deleted the error.

P. 28, line 706: What means without air? Please specify.

We clarified the sentence by adding (line 722/23) that we sealed the plate with parafilm to reduce the air fluctuation.

p. 29, line 722: Did the authors use the Ponceau signal to normalize the Western blot data? More details should be added. Can the authors exclude saturation of the Ponceau signal?

For normalization, we used the Ponceau signal. This was added in line 739. We loaded all blots with the same amount of protein and handled the staining with Ponceau in the same way for all blots. We therefore hope that no saturation occurred.

Hopefully, all points were covered adequate.

We are looking forward to hearing from you.

Sincerely yours,

Gerhard Braus
